# Correlation Between Cardiac Autonomic Dysfunction and Severity of Coronary Artery Lesions in Patients With Chronic Coronary Syndrome

**DOI:** 10.7759/cureus.75756

**Published:** 2024-12-15

**Authors:** Pradyumna K Singh, Tarun Kumar, Bhagya Narayan Pandit, Bhushan Tile, Abhishek Kumawat

**Affiliations:** 1 Cardiology, Atal Bihari Vajpayee Institute of Medical Sciences and Dr. Ram Manohar Lohia Hospital, New Delhi, IND

**Keywords:** cardiac autonomic dysfunction, chronic coronary syndrome (ccs), coronary lesion severity, heart rate recovery, heart rate variability (hrv)

## Abstract

Background

Autonomic dysfunction is associated with adverse outcomes in patients with coronary artery disease. Cardiac autonomic dysfunction parameters such as heart rate variability (HRV) and heart rate recovery (HRR) have been studied individually and have been linked to the presence or likelihood of coronary artery disease. In this study, the cardiac autonomic function was assessed in terms of HRR and HRV. In addition, the association of these parameters with the severity of coronary artery lesions was studied.

Methodology

A total of 100 patients aged more than 18 years who had complaints of typical angina were enrolled in this study. These patients had to undergo the treadmill test for HRR and 24-hour Holter monitoring for HRV. These patients then underwent invasive coronary angiography, and the severity of the lesions was described as single vessel or two/three (multivessel) involvement. The lesions were also grouped as lesions with <70% stenosis and ≥70% stenosis. The association between the severity of the lesions and autonomic dysfunction was studied.

Results

HRR and HRV values were associated with patients with more severe disease. Multivariate regression analysis showed that abnormal HRV was associated with more severe disease; however, abnormal HRR was not directly associated with lesion severity. These autonomic parameters had a negative correlation with the SYNTAX Score.

Conclusions

Our results showed that autonomic dysfunction was associated with more severe coronary artery disease in patients with chronic coronary syndrome. We observed a negative correlation between HRR1 and HRV with the severity of coronary artery disease. Hence, we may try to incorporate these parameters in screening patients with a low likelihood of CAD. Future studies may be planned to see any improvement in cardiac autonomic function after revascularization and its association with outcome.

## Introduction

Coronary artery disease (CAD) is one of the major contributors to death globally. The World Health Organization has reported that India accounts for one-fifth of the burden globally and more so in younger individuals. The Global Burden of Disease study reported a higher age-standardized cardiovascular disease (CVD) death rate of 272 per 100,000 in India compared to the global average of 235 per 100,000 in India [1]. It also reported that CVDs in Indians were described a decade earlier than the western population [2].

Patients with CAD often experience myocardial ischemia and have underlying tissue-level hypoxia. These conditions can disrupt the autonomic nervous system, resulting in deregulation and dysfunction usually manifested by an excessive sympathetic response. This autonomic imbalance is associated with elevated risks of cardiovascular morbidity and mortality. Among the various tests available for autonomic nervous system testing, heart rate recovery (HRR) and heart rate variability (HRV) have been widely used for assessment. Delayed HRR or reduced HRV has been associated with worse outcomes in patients with CAD [3,4]. In 2015, a study by Feng et al [5] concluded that HRV played an important role in estimating the severity of lesions in the coronary artery and autonomic dysfunction. A study conducted by Li et al. in 2016 concluded that reduced HRV predicts CAD in patients with stable angina, independent of traditional risk factors and Framingham risk [6]. Similar results were found in other studies also. Abnormal HRR was also found to be associated with the presence of CAD and in some studies with the severity of CAD. However, not much is known about the status of autonomic nervous function by using a combined assessment of HRR and HRV and their relationship with the severity of coronary lesions in patients with chronic coronary syndrome. Therefore, in the present study, we examined the association of cardiac autonomic dysfunction with the severity of coronary artery lesions.

## Materials and methods

Study design and data collection

This was a single-center, prospective, hospital-based observational study conducted over 12 months from November 2022 to October 2023. Patients were enrolled from the Cardiology outpatient department (OPD) of the hospital during the study period. Routine investigations of these patients were done at the central lab of the hospital. Two-dimensional (2D) echo screening, treadmill test (TMT), and 24-hour Holter were done at the Non-invasive Cardiology lab of the Cardiology Department. Coronary angiography was done at the Catheterisation Laboratory of the institute.

Sample size

According to the study conducted by Chen et al. [7] in 2018, the correlation between abnormal HRR1 and root mean square successive difference (RMSSD) values was found to be around -0.276. With an alpha-error (α) of 5% and 80% power of study (1-β), the sample size was calculated to be 95. However, 100 patients were enrolled in the study. The sample size was calculated using the following formula: sample size (n) = (Z_1-β_ + Z_1-α/2_)^2^ / { r^2^/ (1 -r^2^)}.

Participants

Consecutive patients coming to OPD, who were 18 years or above and had complained of angina which met the three criteria of constricting discomfort in front of the chest or neck, jaw, shoulder, or arm, precipitated by physical exertion and was relieved by rest or nitrates within five minutes [8]. Patients who had previous percutaneous coronary intervention or coronary artery bypass graft, acute myocardial infarction, atrial fibrillation, atrial flutter or ventricular ectopics or high-grade arteriovenous block, New York Heart Association Class IV heart failure, chronic kidney disease, patients who have an ongoing fever or systemic infection, stellate ganglion blockade or are a known case of primary autonomic dysfunction were not included. Only after both the inclusion and exclusion criteria were met patients were enrolled in the study.

Ethical approval

The study was approved by the Institutional Ethics Committee, Atal Bihari Vajpayee Institute of Medical Sciences and Dr. Ram Manohar Lohia Hospital, New Delhi (approval number: TP (DM/Mch) 31/2022)/ IEC/ABVIMS/RMLH/1107) in accordance with the GCP-CDSCO/New Drugs and Clinical Trial rules 2019/ICMR/ (latest amendments) guidelines/ICH-GCP. After explaining the study goals to the patients, written informed consent was obtained from all the participants before enrolling in the study.

Methodology

Once patients were enrolled in the study, history was recorded in their proforma. Clinical examination and routine laboratory investigations including complete blood count, kidney function test, liver function test, fasting and postprandial blood sugar, lipid profile, thyroid profile, a standard 12-lead electrocardiogram (ECG), and a 2D transthoracic echocardiography were acquired from all patients. TMT was done in the department on Norav-ECG 1200 treadmill machine equipped with PC-ECG software 5.8X (Norav Medical GmbH, Wiesbaden, Germany) with continuous ECG monitoring according to the American College of Cardiology/American Heart Association 2002 guideline update for exercise testing [9]. The Bruce protocol was used. Blood pressure was measured and recorded at rest, at the end of each stage, at peak stress, and at the recovery stage until six minutes after exercise. HRR was calculated by subtracting the heart rate values at the first to the fifth minute of the recovery phase from the peak heart rate. HRR1 ≤24 beats/minute and HRR2 ≤42 beats/minute were considered abnormal [10]. Twenty-four-hour Holter Monitoring was done by Philips Holter 1810 with Zymed algorithm (Philips Medical System, USA). HRV is defined as the beat-to-beat variation in time of consecutive heartbeats expressed in normal sinus rhythm on ECG recordings, ranging from a few minutes to 24 hours. HRV parameters were based on the standards of the European Society of Cardiology and the North American Society of Pacing and Electrophysiology [11]. Time-domain HRV parameters included the standard deviation of NN intervals (SDNN) and RMSSD. Coronary angiographic data were recorded with emphasis on the involvement of the number of coronary arteries and the severity of coronary artery lesions. These coronary angiography reports were examined by two cardiologists in the department. The SYNTAX Score was calculated using SYNTAX Score calculator software (https://syntaxscore.org/calculator/syntaxscore). Statistical analysis was conducted to assess the association between the time-domain HRV parameters and HRR with the severity of coronary artery lesions on angiography.

Study outcomes

The primary aim of the study was to analyze the correlation between cardiac autonomic variables and the severity of the coronary artery lesions in terms of the number of vessels involved, degree of stenosis, and SYNTAX I score.

Statistical analysis

Descriptive statistical analyses, including mean and standard deviation for continuous variables and count and percentage for categorical variables, will be performed. Spearman Pearson correlation was used to determine the correlation between HRR and HRV. Logistic regression was used to determine the significant risk factors of coronary artery lesions. All the reported p-values were two-sided, and p-values <0.05 were considered to indicate statistical significance. All data entries and statistical analyses were performed by using SPSS® Version 25.0 software (IBM Corp., Armonk, NY, USA).

## Results

In the present study, the majority of patients were in the age group of 51-60 years. The mean age of the study population was 51.8 ± 7.1 years, ranging from 35 to 65 years of age. The mean age of the patients with multivessel involvement was 55.7 ± 6.2 years. Similarly, patients with coronary artery lesions of more than 70% had a mean age of 54.2 ± 7.6 years. Advanced age was associated with abnormal HRR1 and abnormal SDNN and had a lower mean value of RMSDD. The majority of the population was male at 75% and the remaining 25% were female. Overall, 68% of male patients had underlying CAD, with 56.8% having single-vessel involvement and the remaining 43.2% having multivessel involvement. Similarly, 45.2% of male patients had >70% severity of coronary artery lesions. Among female patients, 60% patients had underlying CAD. About 86.6% had single-vessel involvement. Hence, the overall prevalence of CAD and more severe disease was seen in male patients. Regarding comorbidities, 40% of patients were hypertensive, 23% had diabetes mellitus, 48% had dyslipidemia, 27% had a family history of CAD, and 9% were hypothyroid. The majority of the patients with these comorbidities were male’ however, all patients with hypothyroidism were females. Diabetes and dyslipidemia were found to be significantly associated with multivessel involvement (p < 0.05). Overall, 65.2% of patients with diabetes had multivessel involvement. Similarly, 45.8% of patients with dyslipidemia had multivessel involvement. Patients with diabetes and dyslipidemia also had increased severity of coronary artery lesions. Overall, 30% of patients were found to be smokers and 13% were alcoholics. About 63.3% of patients who were smokers had some form of underlying CAD with predominant involvement of a single coronary artery and coronary artery stenosis <70%. Moreover, 38.4% of patients who were alcoholic had multivessel involvement and 53.8% of patients had stenosis of >70%. We observed that 89% of patients had an ejection fraction (EF) >40% and the remaining patients had about <40% and regional wall motion abnormality. Further, 81.8% of patients with EF <40% had CAD and 55.5% of these patients had more than 70% involvement of the coronary artery.

Based on the number of vessels involved, 34% of our patients had multivessel involvement, 24% had involvement of a single vessel, and the remaining 42% had normal coronaries. Similarly, 31% of patients had stenosis >70%, 27% had 51-70% stenosis, and the remaining had normal coronaries. In our study, 33% of patients had abnormal HRR1, with 54.5% of these patients having multivessel involvement and 48.5% of these patients having >70% severity of coronary artery lesion. Both of these findings were significant with p-values <0.05. We observed that 31% of patients had abnormal SDNN, of whom 70.9% of patients had multivessel involvement. Similarly, 61.2% of the 31 patients had >70% coronary artery stenosis. These associations were significant (p < 0.001).

The clinical profile of patients with a degree of coronary artery stenosis shows that diabetes, dyslipidemia, abnormal HRR1, abnormal HRR2, and SDNN were significantly associated with severity >70% (p < 0.05) (Table 1). The mean RMSDD of 52.6 ± 18.1 ms was significantly lower than other groups with stenosis <70%. The mean SYNTAX score was also higher than other groups for stenosis >70%. Table 1 shows the distribution of demographic features and cardiac autonomic parameters among the group of patients with the categorized groups of degree of stenosis.

**Table 1:** Association between patients’ clinical profile and severity of coronary artery disease (based on the degree of coronary stenosis). HRR: heart rate recovery; RMSSD: root mean square of successive differences; SDNN: standard deviation of NN intervals; EF: ejection fraction

Clinical profile	Degree of coronary stenosis			Total	P-value
	Normal	≤70%	>70%		
Mean age (years)	50.5 ± 6.1	50.8 ± 7.2	54.2 ± 7.6	51.8 ± 7.1	0.061
Male	22	29	24	75	0.205
Female	12	6	7	25	
Hypertension	13	12	15	40	0.489
Diabetes	3	7	13	23	0.006
Dyslipidemia	8	17	23	48	<0.05
Smoking	7	15	8	30	0.108
Alcohol	2	4	7	13	0.128
EF (<40%)	2	4	5	11	0.417
Abnormal HRR1	8	9	16	33	0.029
Mean HRR1	26.8 ± 3.9	26.5 ± 3.7	23.8 ± 4.8	25.8 ± 4.3	<0.05
Abnormal HRR2	5	6	14	25	<0.05
Abnormal SDNN interval	5	7	19	31	<0.05
Mean SDNN interval	117.7 ± 20.5	117.1 ± 20.0	92.2 ± 23.6	109.6 ± 24.2	<0.05
Mean RMSSD	72.1 ± 11.7	68.4 ± 15.2	52.6 ± 18.1	64.7 ± 17.1	<0.05
Mean SYNTAX score	0.00 ± 0.00	6.5 ± 6.8	17.8 ± 11.1	7.8 ± 10.3	<0.05

The clinical profile of patients with the number of vessel involvement showed that multivessel involvement was significantly associated with age, diabetes, dyslipidemia, abnormal HRR1, abnormal HRR2, and reduced HRV (all p < 0.05). The mean RMSDD values for patients with multivalvular involvement (54.9 ± 18.1 ms) were lower in comparison to the mean values for single vessels (66.8 ± 17.3) and normal vessels (71.6% ± 12.2%). The mean SYNTAX score was also higher for multivessel involvement. Table 2 shows the baseline characteristics of patients with normal, single, and multivessel involvement.

**Table 2:** Association between patients’ clinical profile and the severity of coronary artery lesions (based on th number of vessels involved). HRR: heart rate recovery; RMSSD: root mean square of successive differences; SDNN: standard deviation of NN intervals

Clinical profile	Severity of coronary artery lesion			Total	P-value
	Normal	Single vessel	Multiple vessel		
Mean age (years)	50.5 ± 6.0	48.5 ± 7.9	55.7 ± 6.2	51.8 ± 7.1	<0.05
Male	24	29	22	75	0.095
Female	10	13	2	25	
Hypertension	14	9	17	40	0.324
Diabetes	4	4	15	23	0.001
Dyslipidemia	12	13	22	48	0.010
Smoking	11	10	9	30	0.359
Alcohol	3	5	5	13	0.264
Abnormal HRR1	9	6	18	33	0.009
Mean HRR1	27.2 ± 3.9	26.5 ± 3.1	23.5 ± 4.6	25.8 ± 4.3	
Abnormal HRR2	6	3	16	25	<0.05
Abnormal SDNN Interval	6	3	22	31	<0.05
Mean SDNN interval	118.6 ± 19.8	116.7 ± 22.2	93.5 ± 22.7	109.6 ± 24.2	<0.05
Mean RMSSD	71.6 ± 12.2	66.8 ± 17.3	54.9 ± 18.1	67.8 ± 17.1	<0.05
Mean SYNTAX score	0.00 ± 0.00	6.4 ± 2.2	18.4 ± 10.9	7.8 ± 10.3	<0.05

Multiple regression analyses of HRR1 and SDNN with the severity of coronary artery lesions showed that abnormal SDNN is significantly associated with multivessel involvement of coronary arteries (Table 3). Abnormal HRR1 was not observed to be significantly associated with multivessel involvement on multiple regression analysis.

**Table 3:** Multiple logistic regression analysis of HRR1 and SDNN with the severity of disease. HRR: heart rate recovery; SDNN: standard deviation of NN intervals

Severity of coronary artery lesions (based on vessels involved)		P-value	Standard error	Exp(B)	95% CI for Exp(B)	
					Lower bound	Upper bound
Abnormal HRR1	Multiple vessel	0.659	0.648	1.332	0.374	4.743
	Single vessel	0.640	0.668	1.366	0.369	5.064
Abnormal SDNN	Multiple vessel	0.001	0.658	9.444	2.599	34.320
	Single vessel	0.703	0.840	0.726	0.140	3.768

**Figure 1:**
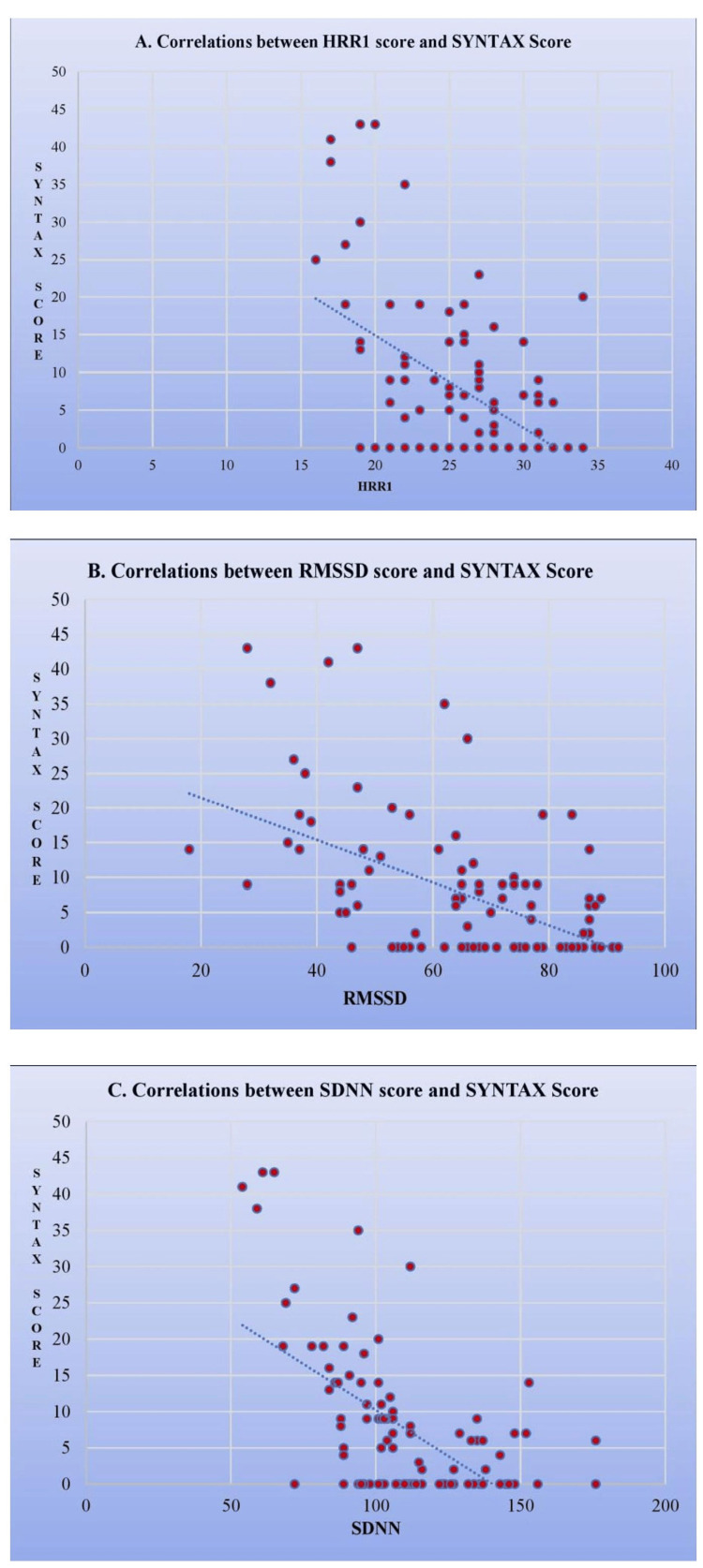
Graphs showing the correlation between (A) HRR1, (B), RMSSD, and (C) SDNN and the severity of coronary lesions (SYNTAX Score). HRR: heart rate recovery; RMSSD: root mean square of successive differences; SDNN: standard deviation of NN intervals

The individual scatter plots of HRR1, SDNN, and RMSDD with SYNTAX score showed a negative correlation, each with a correlation coefficient of -0.508, -0.595, and -0.507, respectively (Figure 1).

**Figure 2:**
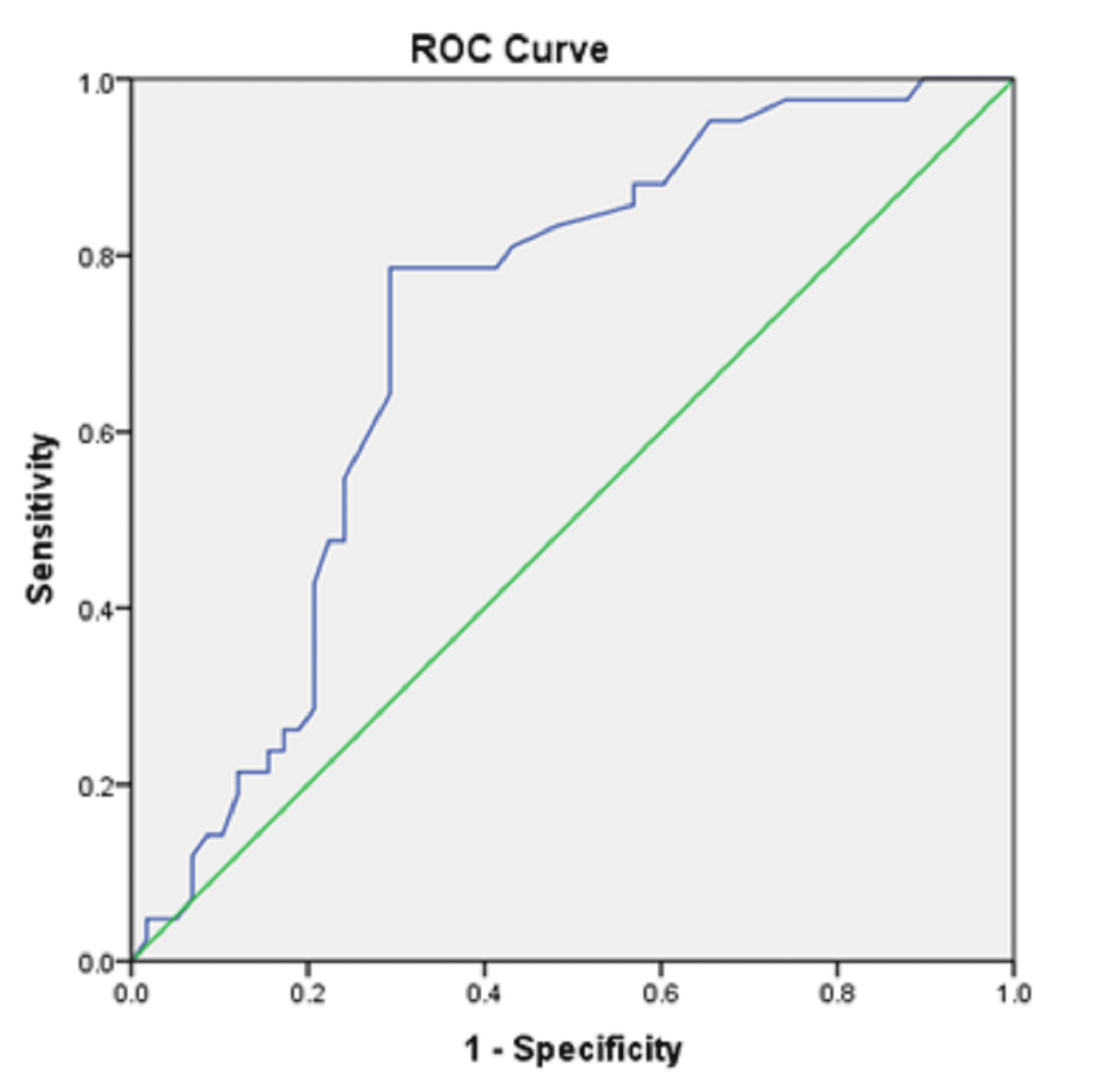
ROC curve of SDNN score for predicting the severity of coronary artery lesions (number of vessels involved). ROC: receiver operating characteristic; SDNN: standard deviation of NN intervals

In our study, the receiver operating characteristic (ROC) curve (Figure 2) when plotted for SDNN interval showed a sensitivity of 78.6% and specificity of 70.6% for a cut-off value of 106.5 for predicting multivessel involvement of coronary arteries.

**Figure 3:**
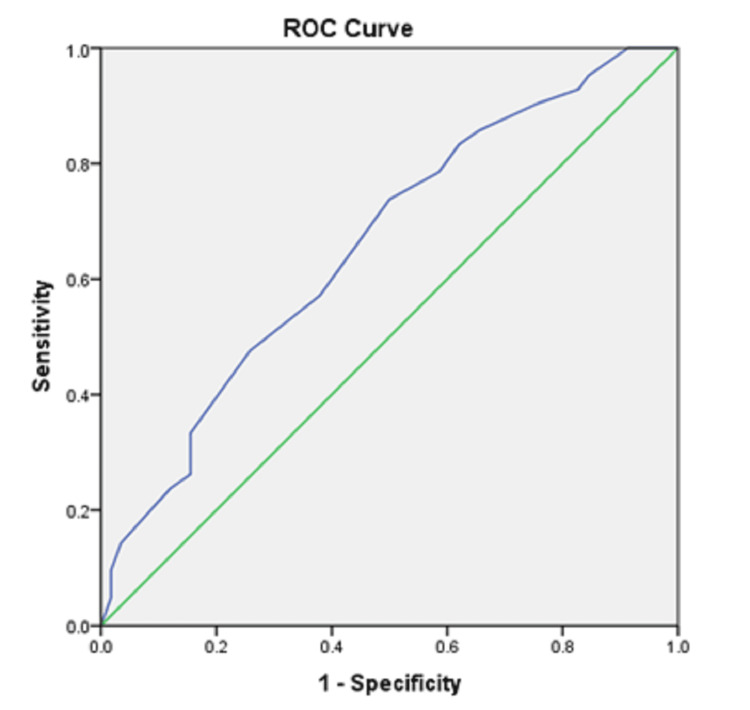
ROC of HRR1 for predicting the severity of coronary artery lesion (number of vessels involved). ROC: receiver operating characteristic; HRR: heart rate recovery

Similarly, for HRR1, the ROC curve (Figure 3) estimates a sensitivity of 57.1% and specificity of 62.1% for a cut-off value of 26.5 for predicting multivessel involvement.

## Discussion

Our study used time-domain HRV parameters and HRR at one minute as markers of abnormal cardiac autonomic activity. We studied the correlation between abnormal SDNN and HRR1 with the severity of CAD. We found that abnormal HRV and HRR1 were associated with multivessel involvement and a higher degree of stenosis. This is consistent with the results of other studies [9]. Similar findings were also reported by Feng et al. [5] where abnormal HRV was related to the severity of stenosis, multivessel involvement, and left CAD. Our results are also supported by the study by Pivatelli et al. [12], where it was seen that there was significantly reduced parasympathetic activity in patients with stable CAD. Reduced parasympathetic activity is represented by abnormal HRR after TMT [13]. Similarly, abnormal HRV is due to reduced parasympathetic and increased sympathetic influence over the sinus node activity.

We found that 31% of patients had abnormal SDNN, of whom 22 (70.9%) patients had multivessel involvement, about 10% of patients had single-vessel disease, and the remaining 19% had normal coronaries. This finding was significant with a p-value <0.05. Our study also showed that mean RMSDD was significantly lower in patients with multivessel involvement and severity of coronary artery involvement in terms of mean SYNTAX score and >70% stenosis was significant with p-values <0.05. The mean RMSDD for multivessel involvement was 54.9 ± 18.1 ms, for single vessels was 66.8 ± 17.3 ms, and for those with normal coronaries was 71.6 ± 12.2 ms. The SDNN was also significantly lower for the groups with more severe disease which indicates a positive correlation between worsening cardiac autonomic function and severity of CAD. Studies by Chen et al. [7], Feng et al. [5], and Pawalak-Bus et al. [14] reported a lower value of RMSDD for severe group CAD but it was not significant. Our study showed a negative correlation between HRV parameters (SDNN and RMSDD) and the number of vessel involvement. Our study also showed a significant association between abnormal SDNN and degree of stenosis >70% (p < 0.001). Overall, 19 out of 31 patients with abnormal SDNN had severity of more than 70%. The values of RMSDD were also significantly lower in patients with >70% stenosis with a p-value <0.05. These findings suggest that there is impaired parasympathetic activity proportionate to the severity of coronary artery lesions. These results are similar to previous studies, which showed HRV can be used to detect myocardial ischemia in subjects who were not diagnosed with CAD, and reduced values of time-domain and frequency-domain HRV parameters such as LF, HF, SDNN, and RMSSD could predict the presence of obstructive CAD [15,16].

Our study showed that abnormal HRR values at one and two minutes were significantly associated with the number of vessel involvement and degree of coronary vessel involvement. Multivessel involvement was seen in 54.5% of patients with abnormal HRR1 and 72% of patients with abnormal HRR2 had multivessel involvement. Similarly, abnormal HRR1 and abnormal HRR2 were significantly associated with >70% stenosis (p < 0.05). The mean SYNTAX score for multivessel involvement and stenosis >70% in patients with abnormal HRR1 was 18.4 ± 10.9 and 17.8 ± 11.1, respectively. It was higher than the group with a lesser degree of stenosis and single-vessel involvement. On the contrary, most of the previous studies have shown the association of abnormal HRR with the presence of CAD and adverse outcomes such as increased mortality or sudden cardiac death. Our findings are supported by a study by Gaffari et al. [17], who reported that abnormal HRR values predicted the severity of stenosis in patients with CAD. In our study, the HRR values were lower for patients with single and multivessel disease compared to those with normal coronaries. In the study by Akyuz et al. [18], abnormal HRR predicted the presence of CAD but not the severity of the lesion. They also reported that abnormal HRR (abnormal HRR <21 beats/minute) was 76.1% sensitive in diagnosis CAD, but did not exhibit good specificity (41.3%). Our study also showed a negative correlation between HRR1 And SYNTAX scores. As seen in our study, abnormal HRR1 with a cut-off value of 26.5 is a predictor of coronary artery lesions with a sensitivity of 57.1% and specificity of 62%. Ghaffari et al. [17] used HRR ≤18 beats as abnormal HRR1 in the supine position, and reported the sensitivity and specificity of HRR for detecting CAD to be 48.0% and 83.3%, respectively.

Notably, we found that abnormal HRR1 and abnormal SDNN were associated with the severity of coronary artery lesions. Both these parameters had a lower value for patients with more severe disease and were negatively correlated. Hence, abnormal cardiac autonomic function as measured from non-invasive methods is a predictor of the severity of coronary artery lesions in patients with chronic coronary syndrome.

Limitations

In this study, although we observed a strong association between the cardiac autonomic parameters and the severity of the coronary artery lesions, there are a few limitations. The study had a small sample size and was conducted at a single center which limits the extrapolation of the findings to the general population. Diabetes, which is a well-known cause of autonomic dysfunction, is among the comorbid conditions included and it may have affected the outcome variables; however, care was taken to include patients with type 2 diabetes mellitus with a duration of less than five years.

## Conclusions

Our study showed that patients with chronic coronary syndrome have abnormal cardiac autonomic function as reflected by the abnormal HRR1 and SDNN. The severity of coronary artery lesions was strongly associated with delayed HRR1 and reduced HRV. Among other parameters, abnormal HRR2 and RMSDD were also abnormal. Based on the strong association observed in our study, we may think of incorporating these into our routine screening methods for coronary angiography in patients with chest pain, especially in patients who belong to the low to intermediate likelihood group for CAD, or who do not have classical angina-like symptoms or who have normal results of other non-invasive testing such as TMT or stress echo. However, studies with large sample sizes are required to further establish our findings. Furthermore, future studies should target the impact of revascularization in patients with CAD on cardiac autonomic function that is whether patients who undergo revascularization show improvement in HRV and HRR. These patients can be followed up to monitor adverse outcomes if any as abnormal HRR has been associated with poor outcomes in patients with abnormal HRR. Pharmacological interventions improving the parasympathetic activity may be tried to look for better outcomes in patients with severe disease.
